# New Alcamide and Anti-oxidant Activity of *Pilosocereus gounellei* A. Weber ex K. Schum. Bly. ex Rowl. (Cactaceae)

**DOI:** 10.3390/molecules21010011

**Published:** 2015-12-22

**Authors:** Jéssica K. S. Maciel, Otemberg S. Chaves, Severino G. Brito Filho, Yanna C. F. Teles, Marianne G. Fernandes, Temilce S. Assis, Pedro Dantas Fernandes, Alberício Pereira de Andrade, Leonardo P. Felix, Tania M. S. Silva, Nathalia S. M. Ramos, Girliane R. Silva, Maria de Fátima Vanderlei de Souza

**Affiliations:** 1 Post-Graduation Program in Development and Technological Innovation in Medicines, Health Science Center, Federal University of Paraiba, Campus I, João Pessoa, PB, 58051-900, Brazil; jksmaciel@hotmail.com (J.K.S.M.); yannateles@gmail.com (Y.C.F.T.); temilce@gmail.com (T.S.A.); 2 Post-Graduation Program in Bioactive Natural and Synthetic Products, Health Science Center, Federal University of Paraiba, Campus I, João Pessoa, PB, 58051-900 Brazil; otemberg_sc@yahoo.com.br (O.S.C.); severinogfilho@yahoo.com.br (S.G.B.F.); marianne.guedes@gmail.com (M.G.F.); 3 Health Science Centre, Physiology and Pathology Department, Federal University of Paraiba, Campus I, Cidade Universitária—João Pessoa, PB, 58059-900, Brazil; 4 Department of Agroecology and Agriculture, Center of Agricultural and Environmental Sciences, University of Paraiba State, 351 Baraúnas Street, Campina Grande, PB, 58429-500, Brazil; pedrodantasfernandes@gmail.com (P.D.F.); albericio3@gmail.com (A.P.A.); lpfelix@hotmail.com (L.P.F.); 5 Postgraduate Program in Development and Technological Innovation in Medicines, Department of Molecular Sciences, Rural Federal University of Pernambuco, Campus Dois Irmãos, Recife, PE, 52171-900, Brazil; sarmentosilva@gmail.com (T.M.S.S.); quinathi@gmail.com (N.S.M.R.); girlianeregina@gmail.com (G.R.S.)

**Keywords:** *Pilosocereus gounellei*, 7′-ethoxy-*trans*-feruloyltyramine, anti-oxidant activity

## Abstract

The Cactaceae family is composed by 124 genera and about 1438 species. *Pilosocereus gounellei*, popularly known in Brazil as xique-xique, is used in folk medicine to treat prostate inflammation, gastrointestinal and urinary diseases. The pioneering phytochemical study of *P. gounellei* was performed using column chromatography and HPLC, resulting in the isolation of 10 substances: pinostrobin (**1**), β-sitosterol (**2**), a mixture of sitosterol 3-*O*-β-d-glucopyranoside/stigmasterol 3-*O*-β-d-glucopyranoside (**3a**/**3b**), 13^2^-hydroxyphaeophytin a (**4**), phaeophytin a (**5**), a mixture of β-sitosterol and stigmasterol (**6a**/**6b**), kaempferol (**7**), quercetin (**8**), 7′-ethoxy-*trans*-feruloyltyramine (mariannein, **9**) and *trans*-feruloyl tyramine (**10**). Compound **9** is reported for the first time in the literature. The structural characterization of the compounds was performed by analyses of 1-D and 2-D NMR data. In addition, a phenolic and flavonol total content assay was carried out, and the anti-oxidant potential of *P. gounellei* was demonstrated.

## 1. Introduction

Cactaceae is a family belonging to the order Caryophyllales with 124 genera and about 1438 species [1] distributed throughout American territory in tropical and temperate dry regions with wide occurrence in Mexico and Brazil [2]. The family is remarkable due to the evolution of several adaptations for aridity, its species showing outstanding diversity of growth forms [3].

Caatinga is a dry eco-region located in northeastern Brazil considered the third diversity center of Cactaceae species, where the family plays an important role, mainly due to the use of its species in human and animal foods [4,5]. Furthermore, many species are used in traditional medicine, for example *Nopalea cochenillifera* is used as an anti-inflammatory, diuretic and hypoglycaemic [6] agent and *Cereus jamacaru* is used to treat ulcers and bronchitis [7].

Previous phytochemical studies on Cactaceae species have reported the presence of flavonoids, such as quercetin, rutin and kaempferol, as well as the anti-oxidant activity of several species, e.g., *Opuntia monocantha*, *Opuntia ficus-indica*, *Cereus jamacaru*, *Pilosocereus pachycladus* and *Pilosocereus arrabidae* [8,9]. In plants, this activity is related to the presence of phenolic molecules such as flavonoids, tannins, phenolic acids, anthocyanins and others [10]. The anti-oxidant activity of phenolics is justified by the presence of conjugated double bonds, which provide resonance, and the hydroxyl groups on an aromatic ring [11], which can chelate metals, eliminate free radicals by donating hydrogen, oxidize and induce enzymes to interact and stabilize free radicals [12,13].

The occurrence of nitrogen compounds such as β-phenethylamines, tetrahydroisoquinolines and their derivatives in this family is also well reported [14,15,16]. Three alkaloids have been isolated from the *Neobuxbaumia* genus: salsolidine, carnegine and anhalidine [17].

*Pilosocereus gounellei* A. Weber ex K. Schum. Bly. ex Rowl, popularly known as xique-xique, is an endemic species from the Caatinga region and its cladodes are used by local people as food, cooked or baked to produce flour, cakes and pastries [18,19]. In folk medicine, its roots are used to treat urinary infections and prostate inflammation. Its cladodes are used to treat gastritis [5,20,21].

This work describes the first isolation of secondary metabolites of *P. gounellei* and the evaluation of the anti-oxidant activity of its extracts and methanol fraction. Anti-oxidant activity of Cactaceae species has been reported previously, justifying our investigation of *P. gounellei*’s anti-oxidant potential [8,9].

## 2. Results and Discussion

### 2.1. Identification of Isolated Compounds

**Figure 1:**
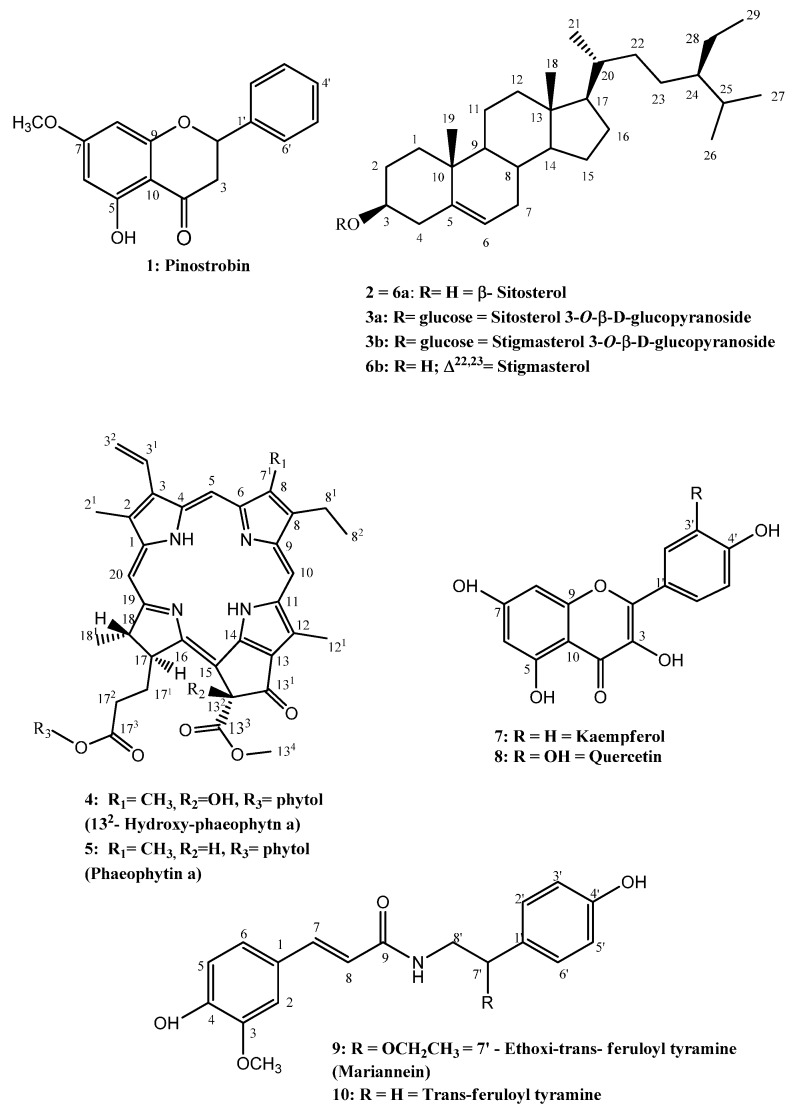
Compounds isolated from *Pilosocereus gounellei*.

**Figure 2:**
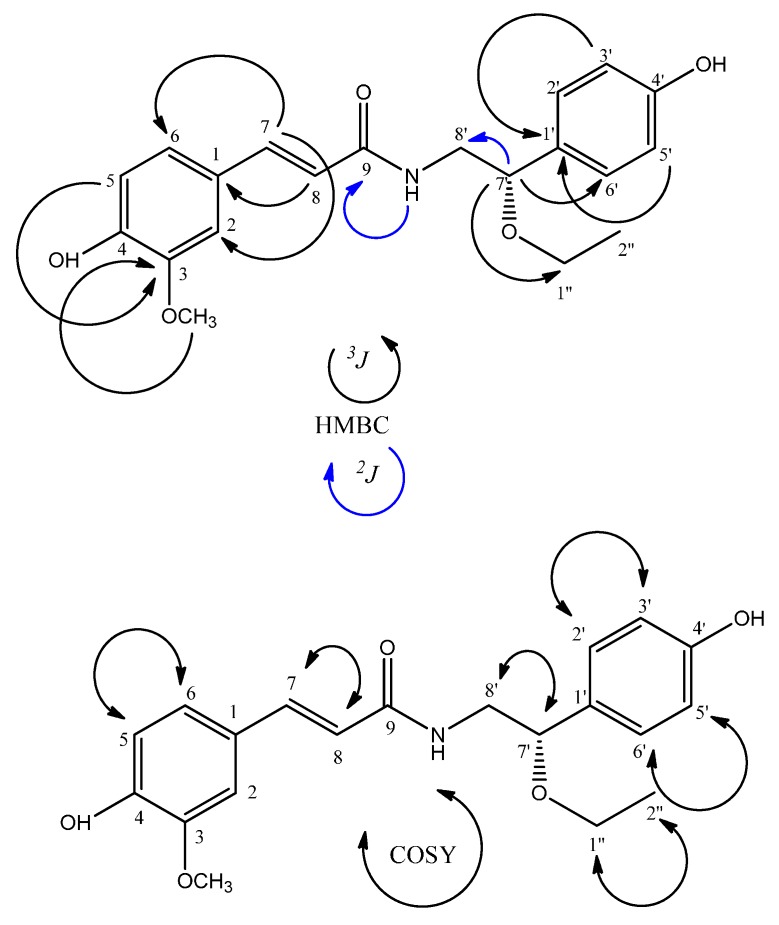
Compound **9** HMBC (*^3^J* and *^2^J*) and COSY correlations.

Chromatographic procedures led to the isolation of 10 compounds from *P. gounellei* (Figure 1 and Figure 2). The isolated compounds were identified by analysis of their 1-D and 2-D NMR data and comparisons with the literature. Compound **1** was isolated as colourless crystals soluble in chloroform and it was identified as the flavonoid pinostrobin [22]. Compounds **2**, **3a/3b** and **6a/6b** were identified as steroids: β-sitosterol, the mixture of sitosterol 3-*O*-β-d-glucopyranoside/stigmasterol, 3-*O*-β-d-glucopyranoside [23] and sitosterol/stigmasterol [24], respectively. These phytosteroids are widespread in plants, being important components of vegetable cell walls and membranes. In addition, they are known as anti-inflammatory agents and precursors of vitamin D [25].

Compounds **4** and **5** were isolated as dark green amorphous solids. The ^1^H- and ^13^C-NMR indicated that the compounds are chlorophyll derivatives. When compared with the literature data, they were identified as 13^2^-hydroxyphaeophytin a (**4**) [26] and phaeophytin a (**5**) [27]. These porphyrinic compounds are derived from chlorophyll a and are widely present in the vegetable kingdom [28]. The substances **7** and **8** were isolated as a yellow amorphous powder and were identified as the flavonoids kaempferol (**7**) [29] and quercetin (**8**) [30]. Flavonoids have great relevance in the pharmaceutical field, displaying anti-oxidant, anti-inflammatory and antimicrobial activities [31], and have been previously reported from several species of the genera *Opuntia* and *Pilosocereus* [8].

The ^1^H-NMR spectrum of compound **9** (Figure 2) showed two doublets (δ_H_ 7.11 and δ_H_ 6.75) integrating for two protons each, suggesting the presence of a *para*-substituted ring. Two doublets in δ_H_ 6.79 (H-5), coupling *ortho*, and δ_H_ 7.11 (H-2), coupling *meta* with H-6 and a double doublet at δ_H_ 6.98 (H-6) coupling *ortho* and *meta* with H-5 and H-2, respectively, indicated the presence of an AMX ring system. The presence of a methoxy group in one aromatic ring was shown by a singlet at δ_H_ 3.79 (3H). The presence of the coumaroyl and tyramine units was suggested by the following signals: a pair of doublets at δ_H_ 6.52 (H-8) and δ_H_ 7.31 (H-7), both with *J* = 16 Hz, characteristic of *trans* olefin protons; an interesting triplet at δ_H_ 8.00, referring to a proton bonded to N [32]; two double doublets at δ_H_ 3.29 (H-8a′) and 3.25 (H-8b′) referring to the methylene carbon adjacent to N and one triplet on δ_H_ 4.27 (H-7′), attributed to a proton on the oximethinic carbon [33]. Other relevant signals in the ^1^H-NMR spectra of **9** were two signals at δ_H_ 3.27 (q) and δ_H_ 1.07 (t) attributed to an ethoxy group attached to an oximethinic carbon whose proton was found at δ_H_ 4.27 (t) [33].

The ^13^C-NMR spectrum showed signals for 20 carbons, supporting the information provided by the ^1^H-NMR spectrum about the presence of the coumaroyl and tyramine portions of compound **9**. The coumaroyl portion was defined by the signals at δ_C_ 165.8 (amide α,β-unsaturated carbonyl) and two signals at δ_C_ 139.46 and δ_C_ 119.01 (the α,β-unsaturated carbons C-7 and C-8). The tyramine portion was confirmed by the signals at δ_C_ 45.61 (CH_2_, C-8′) and an oximethinic carbon δ_C_ 79.6 (CH, C-7′), indicating the position the ethoxy group is attached to [32]. The presence of one ethoxy group in compound **9** was reinforced by the signals at δ_C_ 63.51 (CH_2_, C-1′′) and δ_C_ 15.42 (CH_3_, C-2′′). The ^13^C-NMR spectrum confirmed the presence of two aromatic rings, with δ_C_ 128.07 (C-2′/6′), δ_C_ 115.34 (C-3′/5′) of one AA′BB′ system and δ_C_ 110.9 (C-2), δ_C_ 115.83 (C-5) and δ_C_ 121.82 (C-6) of the AMX system, showing one methoxy group at δ_C_ 55.75 (H_3_CO–C-3) [32,34].

The absence of a correlation for proton δ_H_ 8.00 (t, N-H) with any carbon in the HMQC spectrum, and the correlation shown by the proton at δ_H_ 8.00 (t, 1H) and the carbon at δ_C_ 165.8 (^2^*J*) in the HMBC spectrum demonstrated that the compound possesses an amide carbonyl [32,33,34], being a *trans*-feruloyl derivative [32,33,34]. The HMBC spectrum showed correlations (^3^*J*) that confirmed the presence of the coumaroyl moiety: H-7 with C-2, C-6 and C-9; H-8 with C-1; H-5 with C-3 and C-1. Other correlations in the HMBC spectrum suggest the presence of the ethoxy group and also the *trans*-feruloyl tyramine portion: H-1′′ with C-7′ and H-8′ with C-9 and C-1′. These data allowed identification of compound **9** as 7′-ethoxy-*trans*-feruloyl- tyramine, reported herein for the first time. The COSY spectrum supported the proposed structure by showing correlations between hydrogen (N-H) δ_H_ 8.00 (t) with H-8′; H-8′ and H-7′; H-1′′ and H-2′′ (Table 1). The optical rotation of **9** was found to be −10° (0.01; MeOH) establishing the *S*(−) absolute configuration at the C-7 chiral center [33].

**Table 1:** ^13^C-NMR (100 MHz), ^1^H-NMR (400 MHz), HMQC and HMBC data of 7’-ethoxy-*trans*-feruloyltyramine (**9**) (DMSO-*d*_6_, δ).

Position	HMQC		HMBC	
	δ_C_, Type	δ_H_ (*J* in Hz)	*^3^J* (H C)	*^2^J* (H C)
1	126.62 C	-		
2	110.9 C-H	7.11, d (1.2)	4, 6, 7	3
3	148.03 C	-		
4	148.46 C	-		
5	115.83 C-H	6.79, dd (8.0, 0.72)	1, 3	4, 6
6	121.82 C-H	6.98, dd (8.0, 1.2)	2, 4, 7	5
7	139.46 C-H	7.31, d (16.0)	2, 6 ,9	1, 8
8	119.01 C-H	6.52, d (16.0)	1	9
9	165.87 C	-		
1’	130.78 C	-		
2’/6’	128.07 C-H	7.12, dd (8.0, 1.2)	2’, 6’, 4’, 7’	3’, 5’
3’/5’	115.34 C-H	6.75, dd (8.0, 1.2)	1’, 3’, 5’	4’
4’	157.08 C	-		
7’	79.64 C-H	4.27, t (5.0)	1’’, 2’, 6’	8’
8’	45.61 C-H_2_	3.29 dd, (13.0, 5.9) 3.25, dd (13.0, 5.9)	1’	7’
1’’	63.51 C-H_2_	3.27, q (6.9)		2’’
2’’	15.42 C-H_3_	1.07, t (6.9)		1’’
OMe-3	55.56 C-H_3_	3.79, s	3	
N-H	-	8.0, t (5.0)		9

The molecular formula of new compound **9** was established as C_20_H_23_NO_5_ from a pseudomolecular ion peak at *m*/*z* 358.1706 [M + H]^+^ (calcd. C_20_H_24_NO_5_, 358.1648) indicating the formula C_20_H_23_NO_5_ for the new compound. The corresponding loss of the ethoxy radical fragment was seen in EIMS [M + H]^−^, FTMS as 312.17 (C_18_H_18_NO_4_) and the loss of ethanol was seen in EIMS [M + H]^−^ as 310.17, confirming the proposed structure of compound **9**. The hypotheses that compound **9** is an artefact was eliminated based on previous studies that described the isolation of *trans*-feruloyltyramine and its derivatives by several different extraction methods [33,35]. Liang *et al.* performed the extraction of eight nitrogenated substances from *Portulaca oleracea* using microwave irradiation and different solvents such as dichloromethane, ethyl acetate, methanol, ethanol, ethanol 70%, ethanol 30% and water. The eight isolated substances, including *N*-feruloylnormetanephrine and the *N*-*trans*-feruloyltyramine, were extracted with all the tested solvents, showing that those solvents did not promote the formation of different radicals or artefacts [35].

Compound **10** was obtained as a pale amorphous solid and its spectra showed a structure similar to compound **9**, differing in the absence of signals related to the ethoxy group. Comparisons of the spectral data of compound **10** with compound **9** and literature data allowed identification of compound **10** as *trans*-feruloyltyramine, previously isolated from other plant species [32,33,34]. Compound **10** was shown to possess action against weeds, improvement of seed germination [34] and anti-inflammatory activity by inhibiting COX enzymes [36].

### 2.2. Total Phenolic, Total Flavone and Flavonol Contents and Anti-Oxidant Activity of Extracts and Methanolic Fraction from P. gounellei 

The values of total phenolic, flavone and flavonol contents and anti-oxidant activity (DPPH and ABTS) of ethanol extracts of stems, roots, flowers, fruits and methanol fraction are shown in Table 2. The fruit extract showed the best antiradical activity in the ABTS test (IC_50_ = 10.4 ± 0.24) and the flower extract showed the lowest anti-oxidant activity (IC_50_ = 76.9 ± 0.61); the cladode extract showed a slightly higher activity (IC_50_ = 62.4 ± 0.44) than the flower extract. The scavenging activity of free radicals of the roots extract showed an activity (IC_50_ = 41.6 ± 1.06) similar to the methanol fraction (IC_50_ = 40.9 ± 0.69). Thus, we can classify the activity of extracts and methanol fraction of *P. gounellei* (*Pg*) as follows: *Pg* fruits > MeOH fraction = *Pg* roots > *Pg* cladodes > *Pg* flowers. In the DPPH test, the fruit extract showed the best anti-oxidant activity (IC_50_ = 11.3 ± 0.12), followed by the root extract (IC_50_ = 102.1 ± 1.49). The methanol fraction (IC_50_ = 130.1 ± 3.02) showed similar activity to the cladode extract (IC_50_ = 136.0 ± 3.48) and the least potent was the flower extract (IC_50_ = 194.3 ± 2.33).

**Table 2:** Total phenolic, total flavones and flavonols contents and DPPH and ABTS free radical scavenging activity.

Samples	Total Phenolics Contents (mg GAE/g ± SEM) ^a^	Total Flavones and Flavonols Contents (mg QE/g ± SEM) ^b^	Free-Radical Scavenging Activity (IC_50_) ^c^	
			DPPH (μg/mL ± SEM)	ABTS˙^+^ (μg/mL ± SEM)
*Pg* Cladodes	45.1 ± 2.50 ^1^	12.625 ± 1.08 ^1^	136.0 ± 3.48 ^1^	62.4 ± 0.44 ^1^
*Pg* Methanol Fraction	59.0 ± 2.39 ^2^	13.667 ± 0.833 ^1^	130.1 ± 3.02 ^1^	40.9 ± 0.69 ^2^
*Pg* Roots	61.1 ± 1.03 ^2^	4.920 ± 0.550 ^2^	102.1 ± 1.49 ^2^	41.6 ± 1.06 ^2^
*Pg* Flower	43.5 ± 2.16 ^1^	8.460 ± 0.550 ^3^	194.3 ± 2.33 ^3^	76.9 ± 0.61 ^3^
*Pg* Fruits	127.9 ±1.67 ^3^	2.417± 0.417 ^4^	11.3 ± 0.12 ^4^	10.4 ± 0.24 ^4^
Ascorbic acid	-	-	3.6 ± 0.06 ^5^	-
Trolox	-	-	-	5.0 ± 0.25 ^5^

All values are mean ± S.E.M (*n* = 3); ^a^ GAE = Gallic acid equivalent per gram of sample; ^b^ QE = Equivalent quercetin per gram of sample; ^c^ Value defined as the concentration of sample that scavenged 50% of the DPPH or ABTS**˙^+^**. There are no significant differences among values marked with the same superscript numbers in individual columns.

A correlation has been shown between anti-oxidant activity and total phenolic content in natural products, especially between extracts with the two highest values. The fruit extract showed a greater amount of phenolics (127.9 ± 1.67 mg GAE/g) and also showed the greater anti-oxidant activity in DPPH (IC_50_ = 11.3 ± 0.12) and ABTS (IC_50_ = 10.4 ± 0.24) test. The flower extract presented the lowest anti-oxidant activity in DPPH (IC_50_ = 194.3 ± 2.33) and ABTS (IC_50_ = 76.9 ± 0.61) tests as well as the lowest phenolic content (43.5 ± 2.16). When analysing the other extracts and methanol phase of the plant, a sequence of linear correlations between total phenolic content and radical scavenging activity was not observed.

Among all tested samples, only the fruit extract has sufficient anti-oxidant activity to be considered for nutraceutical use. According to the anti-oxidant activity index (AAI), all extracts and phases of *P. gounellei* showed an index lower than 0.5, thus presenting poor activity. Surprisingly, the fruit extract presented an AAI value of 2.01, which is considered as very strong.

**Figure 3:**
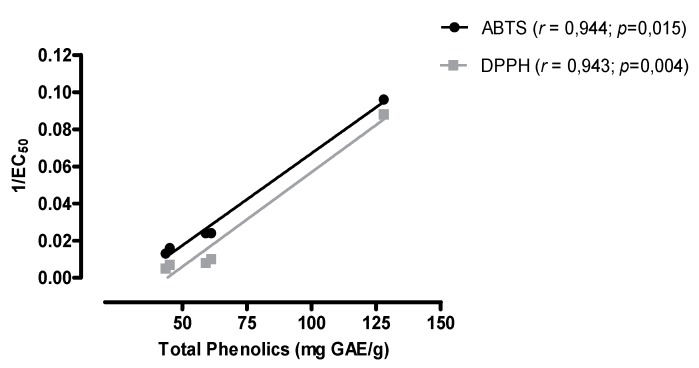
Correlation among the total phenolic content and the antiradical activity DPPH and antiradical activity ABTS˙^+^ of ethanol extracts and methanol fraction of *P. gounellei*.

Many studies have reported the relationship between total phenolics assay results and anti-oxidant activity [37]; our study confirms these findings. The relationships between the total phenolic content and the antiradical activity of DPPH (1/EC_50_), and antiradical activity of ABTS**˙**^+^ (1/EC_50_) are shown in Figure 3. The Pearson correlation coefficients (*r*) of these plots were approximately 0.943 for the DPPH assay and 0.944 for the ABTS assay. This result suggests that 94% of the anti-oxidant capacity of extracts and methanol fraction from *P. gounellei* is due to the contribution of phenolic compounds. It is interesting to mention that there was no inverse correlation between the total phenolic content and the anti-oxidant activity by the DPPH and ABTS methods and flavones/flavonols content, at least when comparing the extract with the highest anti-oxidant activity, the extract from the fruits. Therefore, it is possible that the anti-oxidant activity may be attributed to other phenolic compounds than flavonols and/or flavones**.**

## 3. Experimental Section

### 3.1. General Procedures

Column chromatography separations (CC) were performed on glass columns packed with silica gel 60 (Merck, Nottingham, UK) 7734 (0.063–0.2 mm particles, 70–230 mesh), flash silica (0.04–0.0063 mm particles, 230–400 mesh), Amberlite XAD or Sephadex LH-20. Thin layer chromatography (TLC) was performed on silica gel PF254 plates (Merck, Nottingham, UK). For HPLC experiments, a high-performance liquid chromatograph (HPLC–DAD, equipped with a binary pump (LC-6AD), and diode array detector SPD-M20A (Shimadzu, Kyoto, Japan) and Rheodyne injector (7125) with a 20 μL loop for the analytical column and 500 μL for the semipreparative column were used. An analytical Luna C-18 100A column (250 mm × 4.6 mm × 5 µm, Phenomenex, Torrance, CA, USA), semipreparative Luna C-18 column (250 mm × 21.2 mm × 5 µm, Phenomenex) and Millipore filter membranes (0.45 µm Supelco^®^, Bellefonte, PA, USA) were used.

The solvents used in the chromatographic procedures were p.a. grade: *n*-hexane, dichloromethane, chloroform and ethyl acetate. Methanol HPLC grade (Tedia^®^, Rio de Janeiro, Brazil). Water was obtained from a Millipore^®^ MilliQ system (Millipore, São Paulo, Brazil).

To evaluate the total phenols, total flavones and flavonols content and anti-oxidant activity, the following reagents were used: Folin-Ciocalteu, gallic acid, Trolox (6-hydroxy-2,5,7,8-tetramethylchroman-2-carboxylic acid 97%), ABTS (2,2′-azinobis-(3-ethylbenzothiazoline-6-sulfonic acid) diammonium salt 98%) (Sigma-Aldrich, Sternheim, Germany), ethanol, methanol, sodium carbonate, potassium persulfate, aluminium chloride (Vetec, Rio de Janeiro, Brazil) and Milli-Q water. Samples were solubilized in an ultrasonic bath, 3.5 L (Unic 1600A, Unique, São Leopoldo, Brazil). Readings were performed on Asys HiTech UVM 340 apparatus (São Paulo, Brazil).

Isolated compounds were identified using 1-D and 2-D NMR analysis acquired on the following spectrometers: Varian Oxford (200 MHz), Varian (500 MHz) (Varian, Palo Alto, CA, USA) and Avance III (Bruker, Coventry, UK) using deuterated solvents. The high-resolution mass spectra were obtained using LC-HRMS analysis performed on an Accela 600 HPLC system combined with an Exactive (Orbitrap) mass spectrometer from Thermo Fisher Scientific (Bremen, Germany). EIMS was obtained with a Shimadzu QP-2000 spectrometer (Kyoto, Japan). The ${\left[\alpha \right]}_{\text{D}}^{25}$ 25 °C was determined using a MCP 200 polarimeter (Anton Paar, Saint Laurent, QC, Canada).

### 3.2. Botanical Material

*Pilosocereus gounellei* was collected in Boa Vista City-PB (Brazil) in November 2010. The plant was identified by Prof. Dr. Leonardo Person Felix (CCA/UFPB) and a voucher specimen (15437) was deposited in the Herbarium Prof. Jaime Coelho Morais of the Agricultural Sciences Center (CCA/UFPB).

### 3.3. Extraction and Isolation

The botanical material (cladodes) was dried in an oven with circulating air at 40 °C and ground using a mechanical mill, yielding 5.18 kg of powder, which was macerated with 10 L of EtOH at room temperature, for 72 h. The extraction solution was concentrated in a rotary evaporator at 40 °C, yielding 237.13 g of crude ethanolic extract (CCEE). CCEE (10 g) was submitted to vacuum liquid chromatography (VLC) using silica gel and eluted with hexane (Hex), chloroform (CHCl_3_) and methanol (MeOH) to obtain the corresponding fractions. The chloroform fraction (8.0 g) was chromatographed in a silica column using solvents of increasing polarity: Hex, dichloromethane (CH_2_Cl_2_) and MeOH. The fractions Hex–CH_2_Cl_2_ (3:7), CH_2_Cl_2_ and CH_2_Cl_2_–MeOH (9:1) were combined and chromatographed using the same method yielding 188 fractions that were analysed using TLC. The fractions 35/38 (8.00 mg), after recrystallization, gave pure colourless crystals of compound **1**. The fractions 54/59 (3.51 g) contained compound **2** and the fractions 146/150 (2.72 g) afforded a white powdery precipitate of compound **3**. CCEE (5.00 g) was dissolved in CHCl_3_:H_2_O (1:1) and separated using a separating funnel, yielding an aqueous fraction and a chloroform fraction. A portion of the chloroform fraction (2.0 g) was subjected to successive column chromatography on silica gel, following the methodology previously described, resulting in the isolation of compound **4** (20.50 mg).

CCEE (131.84 g) was submitted to column chromatography using Amberlite XAD as the stationary phase and eluted with H_2_O, MeOH, Hex, ethyl acetate (EtOAc) and acetone. The hexane fraction from XAD was chromatographed using VLC on silica gel 60 with hexane, CH_2_Cl_2_ and MeOH. The hexane fraction was chromatographed on flash silica column yielding the subfractions 28/33 (92.30 mg) that were purified using preparative TLC, eluted with Hex–EtOAc (80:20), to give compound **5** (10.20 mg).

The Hex–CH_2_Cl_2_ (1:1) fraction was chromatographed on silica gel resulting in fractions 13/15 (white crystals), corresponding to **6** (3.51 g). The methanolic fraction from XAD (9.00 g) was chromatographed on Sephadex eluted with MeOH–CH_2_Cl_2_ (7:3) resulting in 33 fractions. Fraction 12 yielded compound **7** (15.00 mg) and fraction 14 yielded compound **8** (12.00 mg), both as yellow powders.

The roots were dried in an oven at 40 °C and ground in a mechanical mill, yielding 1.37 kg of powder that was macerated with 10 L of EtOH at room temperature, for 72 h. The obtained solution was concentrated in a rotary evaporator at 40 °C, resulting in 27.00 g of root crude ethanol extract (RCEE).

To isolate nitrogen-containing compounds, the method described by Souza and Silva [38] was used. The acidified chloroform fraction (ACF, 3.38 g) was chromatographed on a silica column eluted with hexane, EtOAc and MeOH, yielding 159 fractions. The combined fractions 88/114 (580.80 mg) were analysed by HPLC-DAD using a semipreparative column at room temperature. As the mobile phase, Milli-Q water and MeOH were used gradient-wise, and the concentration of the MeOH was increased from 50 to 100% in a 20-min run. The chromatogram showed three peaks, and the one at higher retention time was found to be the major component; thus, it was isolated and identified as compound **9** (8.00 mg).

RCEE (10.00 g) was solubilized in H_2_O and yielded 5.00 g of precipitate. A sample (2.50 g) of it was dissolved in MeOH–CHCl_3_ (1:1) and submitted to filtration on Sephadex LH-20 with MeOH–CHCl_3_ (1:1), resulting in 10 fractions. The subfraction 9/10 (10.00 mg) was proved to be pure by TLC and identified as **10**. The remaining 2.50 g of the precipitate was chromatographed on silica gel 60 column and eluted with hexane, EtOAc and MeOH, yielding the pure fractions 5/7 which corresponded to compound **3**.

### 3.4. Spectral Data

*Pinostrobin* (5-hydroxy-7-methoxy-flavanone, **1**): colorless crystals, molecular formula (C_16_H_14_O_2_) ^1^H-NMR (500 MHz, CDCl_3_): 11.99 (1 H, s, 5-OH), 7.45 ( 5 H, m, H-2’, H-3’, H-4’ e H-5’), 6.06 (1H, d, *J* = 2.5 Hz, H-6), 6.05 (1H, d, *J* = 2.5, H-8), 5.41 (1H, dd, *J* = 13.00 and 3.00 Hz, H-2), 3.79 (3H, s, 7-OMe), 3.07 (1H, dd,17.00Hz e 13.00, H-3), 2.82 (1H, dd, 17.00Hz, 3.00 Hz, H-3). ^13^C-NMR (125 MHz, CDCl_3_): 79.20 (C-2), 43.41 (C-3), 195.73 (C-4), 162.79 (C-5), 95.20 (C-6), 167.80 (C-7), 94.30 (C-8), 164.17 (C-9), 138.39 (C-1’), 126.19 (C2’/C-6’), 128.9 (C-3’, C-4’/C-5’), 55.69 (OCH_3_). 

*β-Sitosterol* (**2**): ^1^H-NMR and ^13^C-NMR data were consistent with the literature [24].

*Sitosterol 3-O-β-d-glucopyranoside/stigmasterol 3-O-β-d-glucopyranoside* (**3**): ^1^H-NMR and ^13^C-NMR data were consistent with the literature [24].

*13^2^-Hydroxy-(13^2^-S)-pheophytin a* (**4**): amorphous green powder ^1^H-NMR and ^13^C-NMR data were consistent with the literature [26].

*Phaeophytin a* (**5**): morphous green powder, ^1^H-NMR and ^13^C-NMR data were consistent with the literature [27].

*β-Sitosterol and stigmasterol* (**6**): ^1^H-NMR and ^13^C-NMR data were consistent with the literature [24].

*Kaempferol* (3,4′,5,7-Tetrahydroxyflavone, **7**): yellow amorphous powder, ^1^H-NMR and ^13^C-NMR data were consistent with the literature [29].

*Quercetin* (3,3′,4′,5,7-Pentahydroxyflavone, **8**): yellow amorphous powder, ^1^H-NMR and ^13^C-NMR data were consistent with the literature [30].

*7’-Ethoxy-trans-feruloyltyramine* (**9**): a pale amorphous solid, molecular formula (C_20_H_23_NO_5_), ^1^H-NMR (δ, 400 MHz, DMSO-*d*_6_) and ^13^C-NMR (δ, 100 MHz, DMSO-*d_6_*): see Table 1. 

*trans-Feruloyltyramine* (**10**): pale amorphous solid, molecular formula (C_18_H_19_NO_4_), ^1^H-NMR (δ, 400 MHz, DMSO-*d_6_*), 7.11 (1H, sl, H-2), 6.78 (1H, d, *J* = 8.0Hz, H-5), 6.98 (1H, d, *J* = 8.0 Hz, H-7), 7.30 (1H, d, *J* = 16Hz, H-7), 6.43, (1H, d, *J* = 15.8 Hz, H-8), 7.00 (2H, d, *J* = 8.0, H-2’/H-6’), 6.68 (2H, d, *J* = 8.0 Hz, H-3’/H-5’), 2.64 (2H, t, H-7’), 3.33 (2H, m, H-8’), 3.79 (3H, s, OCH_3_), 8.01 (1H, t, NH). ^13^C-NMR (δ, 100 MHz, DMSO-*d*_6_) 126.93 (C-1), 111.12 (C-2), 148.37 (C-3), 148.75 (C-4), 116.18 (C-5), 122,09 (C-6), 139.56 (C-7), 119.53 (C-8), 128.19 (C-1’), 130.02 (C-2’/C-6’), 115.67 (C-3’/C-5’), 156.18 (C-4’), 34.27 (C-7’), 41.27 (C-8’), 56.07 (OCH_3_).

### 3.5. Total Phenol Content Assay

The total phenolics content was evaluated by the method of Gulcin *et al.*, [39] with some modifications, using the Folin-Ciocalteu reagent and gallic acid as a positive control. Samples of cladodes, roots, flowers and fruit and the methanolic fraction from *P. gounellei*, from a stock solution of 5 mg/mL, solubilized in EtOH, were transferred to a 1.0 mL Eppendorf tube by adding 20.0 µL of Folin-Ciocalteu reagent, stirring for 1 min. Then Na_2_CO_3_ (60.0 µL, 15%) was added to the mixture and stirred for 30 s. Finally, distilled water (900 μL) was added to give a final concentration of 100 μg/mL. After 2 h, the absorbance of the samples was measured at 760 nm. The concentration of the phenolic compounds was determined as equivalent milligram of gallic acid per gram of sample (mg GAE/g), from the calibration curve constructed with gallic acid standard (2.5 to 15.0 µg/mL), considering the average standard error (SEM). 

### 3.6. Total Flavones and Flavonols Content Assay

The flavones and total flavonols content were determined adapting the methodology described by Mihai *et al.* [40]. Stock solutions (1.0 mg/mL) of extracts from cladodes, roots, flowers and fruits and methanolic fraction from *P. gounellei* were prepared. Each sample solution (400 µL) and methanolic solution of aluminium chloride (200 µL, 2%) were added in a volumetric flask. The final volume was adjusted with the same solvent to 10 mL. Reaction occurred for 30 min in the dark. The reading was performed at a wavelength of 425 nm. The analysis was evaluated in triplicate and the total flavones and flavonols content was determined from the calibration curve constructed with straight line equation of quercetin solutions (1.0 to 40.0 µg/mL) and expressed in equivalent milligrams of quercetin by gram of extract (mg QE/g), considering the average standard error (SEM).

### 3.7. DPPH**˙** Radical Scavenging Activity Assay

The anti-oxidant activity of ethanolic extracts of the cladodes, roots, flowers and methanol fraction from *P. gounellei* was performed against the free radical DPPH following the methodology of Silva *et al.* [41]. Stock solutions were prepared from the extracts and methanol fraction at several concentrations (0.10 to 5.0 mg/mL). Through preliminary analysis, appropriate quantities of stock solutions of the samples and 450 µL of the solution of DPPH**˙** (23.6 mg/mL in EtOH) were transferred to 0.5 mL Eppendorf tubes and the volume was completed with EtOH, following homogenization. Samples were sonicated for 30 min and the amount of DPPH**˙** was recorded on a UV-vis device at a wavelength of 517 nm in a 96-well plate. Ascorbic acid was used as a positive control and all concentrations were tested in triplicate.

The percentage scavenging activity (% SA) was calculated from the equation:

(1) $$\%\;\text{SA}=100\times \frac{\left(Abs_{\text{control}}-Abs_{\text{sample}}\right)}{Abs_{\text{control}}}$$

where *Abs*_control_ is the absorbance of the control containing only the ethanol solution of DPPH, and *Abs*_sample_ is the absorbance of the radical in the presence of the sample or standard ascorbic acid. The anti-oxidant activity index (AAI) was calculated according to Scherer and Godoy [42] as follows:

(2) $$\text{AAI}\;=\;\frac{\text{final concentration of DPPH }\left(\mu \text{g}\cdot {\text{mL}}^{-1}\right)}{{\text{IC}}_{50}\left(\mu \text{g}\cdot {\text{mL}}^{-1}\right)}$$

AAI values below 0.5 indicate low anti-oxidant activity, values between 0.5 and 1.0 indicate moderate activity, values between 1.0 and 2.0 indicate strong activity and AAI values above 2.0 indicate very strong anti-oxidant activity.

### 3.8. Determination of Anti-oxidant Activity against the Radical Cation ABTS**˙**^+^

The determination of anti-oxidant activity from extracts of cladodes, roots, flowers, fruits and methanolic fraction from *P. gounellei* against the radical cation ABTS**˙^+^** was carried out following the methodology described by Re [43] using Trolox as the standard compound. The starting concentrations of the solutions of the samples were 0.1–1.0 mg/mL, with the addition of 450 µL of the radical ABTS**˙^+^** solution to give final concentrations of 2.5–100.0 μg/mL samples. Samples were protected from light and sonicated for 6 min. Absorbance of the samples and the positive control were measured at a wavelength of 734 nm using a microplate of 96 wells. Each concentration was tested in triplicate. The percentage of free radical scavenging activity of ABTS**˙^+^** was calculated by the equation:

(3) $$\%\;\text{SA}=100\times \frac{\left(Abs_{\text{control}}-Abs_{\text{sample}}\right)}{Abs_{\text{control}}}$$

where *Abs*_control_ is the absorbance of the control containing only the ethanol solution of ABTS**^+^** and *Abs*_sample_ is the absorbance of the radical in the presence of the sample or standard ascorbic acid.

The antiradical efficiency was established using linear regression analysis and the 95% confidence interval (*p* < 0.05) obtained using the statistical program GraphPad Prism 5.0. The results were expressed as EC_50_ ± SEM (sample concentration required to eliminate 50% of the DPPH radicals available, plus or minus the SEM).

### 3.9. Statistical Analyses

The results are expressed as the mean ± standard error of the mean (SEM). Analysis of variance (ANOVA one-way and Tukey’s *post hoc* test) were used to evaluate the differences of the means between groups. The antiradical efficiency was established using linear regression analysis. Pearson correlation coefficients (*r*) were used to express correlations and confidence interval of 95% (*p* < 0.05) obtained using the statistical program GraphPad Prism 5.0 (GraphPad Software Inc., San Diego, CA, USA). The results were expressed by sample concentration required to eliminate 50% of the DPPH radicals available, plus or minus the SEM (EC_50_ ± SEM).

## 4. Conclusions 

The phytochemical study of *Pilosocereus gounellei* led to the isolation and identification of 10 compounds: pinostrobin, β-sitosterol, a mixture of β-sitosterol/stigmasterol, 13^2^-hydroxy-phaeophytin a, phaeophytin a, sitosterol 3-*O*-β-d-glucopyranoside/stigmasterol 3-*O*-β-d-gluco-pyranoside, kaempferol, quercetin, the new substance 7′-ethoxy-*trans*-feruloyltyramine and *trans*-feruloyltyramine. The evaluation of anti-oxidant activity from *P. gounellei* demonstrated that the fruit ethanol extract possesses excellent anti-oxidant activity, mainly because of the presence of phenolic compounds reported in the genus and the Cactaceae family.
